# Data mining technique for identifying mortality patterns in a pulmonary tuberculosis cohort

**DOI:** 10.1590/0102-311XEN203124

**Published:** 2026-03-23

**Authors:** Marli Souza Rocha, Valéria Saraceni, Claudia Medina Coeli, Max Leonardo Chacón Pacheco, Rejane Sobrino Pinheiro

**Affiliations:** 1 Instituto de Estudos em Saúde Coletiva, Universidade Federal do Rio de Janeiro, Rio de Janeiro, Brasil.; 2 Ministério da Saúde, Brasília, Brasil.; 3 Secretaria Municipal de Saúde do Rio de Janeiro, Rio de Janeiro, Brasil.; 4 Facultad de Ingeniería, Universidad de Santiago de Chile, Santiago, Chile.

**Keywords:** Tuberculosis, Mortality, Information Systems, Medical Record Linkage, Tuberculose, Mortalidade, Sistemas de Informação, Registro Médico Coordenado, Tuberculosis, Mortalidad, Sistemas de Información, Registro Médico Coordinado

## Abstract

Tuberculosis (TB) deaths occur early, mainly during the two months comprising the intensive treatment phase compared to other causes of death. Understanding the distinct TB mortality patterns provides more effective health care for specific groups. This study aimed to apply a data mining technique to examine whether there are distinct mortality patterns associated with diverse patient profiles within a cohort notified with TB in the city of Rio de Janeiro, Brazil. This was a retrospective cohort study using a probabilistic linkage between the Brazilian Information System for Notifiable Diseases (SINAN) and the Brazilian Mortality Information System (SIM). An association rule data mining technique was applied using the Apriori algorithm to evaluate patterns associated with causes of death. TB deaths were associated with adult men with short survival time. Deaths from AIDS with TB occurred during the treatment period and were more associated with women. In contrast, deaths from AIDS without a mention of TB were more frequent among younger men and Black/Mixed-race adults. Deaths from external causes occurred later, one year after diagnosis, and were related to young men. Deaths from other causes that were linked to longer survivals were associated with older white individuals with higher education. The Apriori algorithm provided the extraction of relevant knowledge from secondary data, such as identifying distinct subgroups of people who can benefit from specific actions, with a consequent positive effect for TB control.

## Abstract (pt)

As mortes por tuberculose (TB) ocorrem precocemente, principalmente durante os dois meses que compõem a fase de tratamento intensivo, em comparação com outras causas de morte. Compreender os padrões distintos das pessoas que morrem devido à TB permite melhor eficácia do atendimento à saúde para grupos específicos. O objetivo foi aplicar a técnica de mineração de dados para explorar se existem padrões distintos de mortalidade associados a perfis diversos de pacientes dentro de uma coorte de indivíduos notificados com TB na cidade do Rio de Janeiro, Brasil. Realizamos um estudo de coorte retrospectivo usando uma ligação probabilística entre o banco de dados de TB do Sistema de Informação de Agravos de Notificação (SINAN) e o Sistema de Informações sobre Mortalidade (SIM). A técnica de mineração de dados foi aplicada usando o algoritmo Apriori para avaliar padrões associados às causas de morte. Mortes por TB estavam associadas a homens adultos com tempo de sobrevivência curto. Mortes por aids com TB ocorreram durante o período de tratamento e foram mais associadas às mulheres. Em contraste, mortes por aids sem menção à TB foram mais frequentes entre homens mais jovens e adultos negros/pardos. Mortes por causas externas ocorreram mais tarde, um ano após o diagnóstico, e estavam relacionadas a homens jovens. Mortes por outras causas ligadas à sobrevivência mais longa estavam associadas aos brancos idosos com Ensino Superior. O algoritmo Apriori permitiu a extração de conhecimento relevante a partir de dados secundários, como identificar subgrupos distintos de pessoas que podem se beneficiar de ações específicas, com um consequente efeito positivo para o controle da TB.

## Abstract (es)

Las muertes por tuberculosis (TB) se producen de forma temprana, principalmente durante los dos meses que comprende la fase de tratamiento intensivo, en comparación con otras causas de muerte. Comprender los distintos patrones de las personas que fallecen por TB permite mejorar la eficacia de la atención sanitaria para grupos específicos. El objetivo del estudio era aplicar una técnica de minería de datos para explorar si existen patrones de mortalidad distintos asociados a diversos perfiles de pacientes dentro de una cohorte de personas notificadas con TB en la ciudad de Río de Janeiro, Brasil. Realizamos un estudio de cohorte retrospectivo utilizando un enlace probabilístico entre la base de datos del Sistema de Información de Agravios de Notificación (SINAN) y el Sistema de Informaciones sobre Mortalidad (SIM). Se aplicó una técnica de minería de datos en reglas de asociación utilizando el algoritmo Apriori para evaluar los patrones asociados con las causas de muerte. Las muertes por TB se asociaron con hombres adultos con un tiempo de supervivencia corto. Las muertes por SIDA con TB se produjeron durante el periodo de tratamiento y se asociaron más con las mujeres. Por el contrario, las muertes por SIDA sin mención de TB fueron más frecuentes entre los hombres más jóvenes y los adultos negros/morenos. Las muertes por causas externas se produjeron más tarde, un año después del diagnóstico, y se relacionaron con los hombres jóvenes. Las muertes por otras causas relacionadas con una mayor supervivencia se asociaron con personas mayores, blancas y con un nivel educativo superior. El algoritmo Apriori permitió extraer información relevante de datos secundarios, como la identificación de subgrupos distintos de personas que pueden beneficiarse de medidas específicas, con un consecuente efecto positivo para el control de la TB.

## Introduction

Tuberculosis (TB) continues to pose a substantial public health challenge. Brazil is one of the 30 countries with the highest rates of TB ^1^. Despite advancements in diagnosis, prevention, as well as effective, free, and widely available treatment provided by the Brazilian Unified National Health System (SUS, acronym in Portuguese), over 80,000 new cases are diagnosed and approximately 6,000 deaths are recorded annually ^2^. This burden places the country, along with Peru, among those with the highest rates of TB in the Americas ^1^.

In recent years, several key health care interventions have been implemented in Brazil to improve TB control. These include the directly observed treatment implemented in the health care system, the expansion of the Rapid Molecular Testing Network for TB (RMT-TB), enhancements to the quality of active TB treatment, the expansion of preventive TB treatment (TPT), and the strengthening of primary health care (PHC) for the early identification of individuals with TB and the improvement of treatment adherence and monitoring ^3^. However, TB control remains unsatisfactory. Cure rates consistently fall short of World Health Organization (WHO) recommendations. Adverse outcomes such as treatment nonadherence and mortality are frequently observed. The death of a TB patient is considered a preventable event, with the WHO’s goal for Brazil by 2035 being a 95% reduction in TB-related fatalities.

Globally and in Brazil, several TB-associated factors have been linked to mortality, including advanced age, low educational level, use of alcohol and other drugs, TB-HIV coinfection, and loss to follow-up ^4^ ^,^ ^5^ ^,^ ^6^ ^,^ ^7^, as well as characteristics of health care services. Health care service-related factors, such as having been treated in an emergency hospital, are also linked to an increased risk of death ^5^. However, it is currently not clear whether or not the factors vary with the causes of death, during and after TB treatment, and can be associated with the burden of TB and HIV in the investigated region ^4^. Some studies in South India (Asia), Spain (Europe), Tanzania (Africa), and Brazil (South America) indicate TB deaths occurring early, mainly during the two months comprising the intensive treatment phase ^6^ ^,^ ^7^ ^,^ ^8^ ^,^ ^9^ ^,^ ^10^. However, such studies are scarce in Brazil.

Understanding the mortality patterns in TB patients and their association with specific risk profiles is essential for guiding public health surveillance, management, and care. This information would facilitate the prioritization of targeted interventions, aiming to enhance health care effectiveness and reduce disease-related fatalities. A study in the same municipality found that the primary underlying causes of death were TB and AIDS ^11^. Among other causes, the most common were neoplasms, circulatory diseases, and respiratory system diseases.

Data mining algorithms are valuable tools for the identification of patterns associated with a variety of health outcomes. When compared to traditional statistical methods, such tools can provide new insights, especially when studying many covariates that yield the dispersion of observations in multiple profiles ^12^. Association rule algorithms are used to extract key patterns from data that can identify patient profiles with different risks of death. Initially used in market basket analysis for identification of items that tend to be purchased together, these algorithms have been increasingly used in health studies ^13^ ^,^ ^14^ ^,^ ^15^ ^,^ ^16^ ^,^ ^17^ ^,^ ^18^. However, there are few TB studies, and those are predominantly for diagnosis ^16^ ^,^ ^18^.

The city of Rio de Janeiro is the second-largest economy in Brazil and has one of the highest TB mortality rates, more than double the country’s average ^2^. In light of this, this seeks to apply a data mining technique to examine whether there are distinct mortality patterns associated with diverse patient profiles within a cohort of individuals notified with TB in the city of Rio de Janeiro. The ability to distinguish specific groups of individuals could facilitate the establishment and prioritization of targeted actions, thereby improving the effectiveness of health care and reducing TB-related mortality.

## Method

### Data sources and study population

This study used a linked database from a previous investigation ^19^. This database was composed of a cohort of individuals with TB reported in the Brazilian Information System for Notifiable Diseases (SINAN, acronym in Portuguese) between 2009 and 2012. The cohort was passively followed for at least one year after notification through record linkage with the Brazilian Mortality Information System (SIM, acronym in Portuguese) database, covering the period from 2009 to 2013. The study population consisted of individuals living in Rio de Janeiro, diagnosed with pulmonary TB, who died between 2009 and 2013. When a person had more than one treatment reported at SINAN, only the most recent record was analyzed because the information was closer to the death. Subjects aged under 15 years of age and those with HIV infection were excluded. Also, records of individuals with HIV/AIDS were also excluded due to their elevated risk of TB mortality, unlike other patient groups. We restricted the analysis to cases of pulmonary TB in adults given their higher prevalence and the unexpected nature of death as an outcome in this specific group.

### Probabilistic record linkage

We carried out a probabilistic record linkage using the patient’s name, mother’s name, sex, date of birth, and address ^20^.

We used 22 blocking steps for indexing the database to improve sensitivity, using a combination of variables, as follows: soundex of the person’s given name; soundex of the person’s last name; sex; year of birth; city of residence; soundex of the mother’s given name, and soundex of the mother’s last name ^21^. We used the person’s full name, mother’s full name, and birth date to compute each pair of records’ match score ^20^.

We empirically defined the match score threshold to classify a pair of records as belonging to the same person. This procedure involved a top-down inspection of record pairs, ordered by their scores, within the OpenReclink environment ^22^. We continued this inspection until we encountered pairs for which we could not confidently determine if they belonged to the same person. These pairs, along with all others with a score from this threshold down to zero, formed a “gray area” of uncertain classification. All pairs within this gray area subsequently underwent clerical review to enhance accuracy, based on the person’s full name, mother’s full name, date of birth, and address. When in doubt, we classified the pair of records as a non-match, adopting a more conservative classification procedure ^23^.

### Apriori algorithm

Association rule data mining is a technique used to discover meaningful patterns and extract valuable new knowledge from large datasets. The Apriori algorithm is a widely used method for this end, as it efficiently identifies all frequent itemsets in a dataset to then form relevant association rules. Each category of a variable is equivalent to an item of the Apriori algorithm. An association rule relates two sets of data (called items) as a conditional sentence IF (X) THEN (Y) (short form X → Y), in which X is a set of binary items joined by the condition “and”, called antecedent, and Y is a set of binary items, disjunctive from X, also joined by the condition “and”, called consequent ^12^. Y is defined as a fixed set of items (an example would be the items represented by the treatment outcomes), whereas X are sets formed by combinations of all items x_i_ in set X = {x_1_, x_2_, ..., x_n_}. As an example, these items would be the factors that can determine the outcomes.

The purpose of association rules is to find all possible sets of items X that come together with those of set Y ^12^. Different metrics are used to assess the rules’ frequency and strength ^24^. The support of a rule is the proportion of items that appear in the antecedent and consequent concomitantly, concerning the total records of the database. It can also be defined as the frequency of patterns in the database, for instance, the proportion of a certain patient profile that died from a specific cause in the total number of records in the database.

The confidence of a rule measures the proportion of people who died from a certain cause (consequent), among a certain profile of individuals (antecedent) ^18^. Lift is a measure that assesses the dependency between items in a rule. Lift evaluates the degree to which the occurrence of one item leads to the occurrence of the other ^14^, that is, it indicates dependence, the more frequent Y becomes when X occurs, given an association rule X - > Y. For instance, the lift of a rule indicates how much greater is the occurrence of a given profile with a cause of death, compared to the expected, if the given profile was independent of that cause of death.

Minimum support is required to assure the statistical significance of the generated standards, restricting the number of rules originated by the algorithm. High confidence values are vital to ensure a high relationship between the items analyzed. The more relevant rules are those that have a high lift value ^25^.

### Variables and items used

The antecedent items were defined by several variables, including sociodemographic aspects, the status of diagnostic laboratory tests for TB and HIV, the type of health care service, and time until death (Box 1). The laboratory test variable had categories for whether the test was performed, not performed, or if the result was not registered in the database. The consequents were grouped according to the underlying cause of death and categorized into deaths from TB, external causes, AIDS without a mention of TB, and AIDS with TB, considering the codes of the International Classification of Diseases - 10th revision (ICD-10) registered in the primary cause: A15-A19, V01-Y98, B20.1-B24, and B20.0, respectively. Deaths from other causes were all deaths different from those mentioned above.

**Box 1:** Description of the variables and items used in the analysis of tuberculosis (TB) mortality. Rio de Janeiro, Brazil, 2009 to 2012.

CHARACTERISTICS	ITEMS
Age	15-34 years, 35-59 years, 60 years or older
Sex	Female, male
Skin color	White, Black/Mixed-race, Yellow, Indigenous, ignored
Education	0-4 years, 5-8 years, 9-12 years, > 12 years, ignored
Type of entry	New case, relapse, return after default
HIV test result	Negative, in progress, not tested
Sputum smear test	Tested, not tested
Type of health care facility	Family Health Strategy, Municipal Health Care Center, TB clinic, hospital
Survival time (time elapsed from the date of diagnosis in SINAN to the date of death in SIM)	Notified after death, 0-60 days, 61-180 days, 181-365 days, greater than 365 days
Underlying cause of death	TB (A15-A19), AIDS with TB (B20.0), AIDS without TB (B20.1-B24), external causes (V01-Y98), other causes (other ICD-10 codes)

ICD-10: International Classification of Diseases - 10th revision; SIM: Brazilian Mortality Information System; SINAN: Brazilian Information System for Notifiable Diseases.

We included the HIV test variable to account for individuals whose underlying cause of death was AIDS but who had no recorded HIV test result. This procedure enabled us to identify possible undetected HIV patients at the time of notification of TB. No filtering was applied for prior causes of death to ensure a complete list of all potential recorded causes.

Survival time was defined as the number of days elapsed from the date of diagnosis in SINAN to the date of death registered in SIM.

### Data analysis

We defined a minimum support of 0.5% to enable the generation of much less common rules. We eliminated redundancies considering confidence ^24^ and also rules with a lift below 1.1. Three experts in TB surveillance convened to select the optimal rules, based on both predefined metrics and their professional expertise. The selection criteria prioritized confidence and lift, followed by support or the more informative rule, as indicated by the specialists’ assessment.

The analyses used the R statistical package version 3.3.1 (http://www.r-project.org) with the libraries “arules” ^26^ ^,^ ^27^ and “arulesViz” ^28^. The probabilistic record linkage used the OpenReclink software ^22^.

## Results

The analysis included 1,819 individuals diagnosed with pulmonary TB who died during the follow-up of the cohort. Table 1 shows the absolute and relative frequencies of the 36 items from 10 variables. Approximately three-quarters were new TB cases, male, and about half were aged 35 to 59 years, had less than nine years of education, Black/Mixed-race skin color, did not undergo an HIV test, and underwent sputum smear testing for TB diagnosis. Almost half of the deaths occurred within the treatment period, with the majority (30.6%) in the first two months after diagnosis. Most deaths were due to other causes (52%), followed by TB (36%) and AIDS (3.5%) (Table 1).

**Table 1:** Absolute and relative frequencies of items used in the analysis of tuberculosis (TB) mortality. Rio de Janeiro, Brazil, 2009 to 2012.

Items	n	%
Sex		
Female	451	24.8
Male	1,368	75.2
Age (years)		
16-34	367	20.2
35-59	786	43.2
60 or older	666	36.6
Education (years)		
0-4	464	25.5
5-8	409	22.5
9-12	186	10.2
More than 12	66	3.6
Ignored	694	38.2
Skin color		
White	699	38.4
Yellow	10	0.5
Indigenous	3	0.2
Black/Mixed-race	902	49.6
Ignored	205	11.3
HIV test		
Negative	758	41.7
In progress	76	4.2
Not tested	985	54.1
Type of entry		
New case	1,442	79.3
Relapse	138	7.6
Return after default	239	13.1
Type of health care facility		
Family Health Strategy	67	3.7
Municipal Health Care Center	874	48.1
TB clinic	119	6.5
Hospital	755	41.5
Others *	4	0.2
Sputum smear test for TB diagnosis		
Tested	962	52.9
Not tested	857	47.1
Survival time		
Notified after death (days)	42	2.3
0-60	557	30.6
61-180	341	18.7
181-365	228	12.6
Over 365	651	35.8
Underlying cause of death		
TB	655	36.0
AIDS without TB	26	1.4
AIDS with TB	38	2.1
External causes	155	8.5
Others	945	52.0
Total	1,819	100.0

* Including laboratories, blood centers, and emergency units.

We generated 3,590 association rules without redundancy: 1,222 for deaths from TB, 73 for deaths from AIDS and TB, 23 for deaths from AIDS without TB, 612 deaths from external causes, and 1,660 deaths from other causes.

Table 2 shows the rules selected for deaths from TB. The rules selected had maximum support of 1.4%, a maximum lift of 2.8%, and maximum confidence of 100%. The items male and short survival periods are noted. The rules with higher confidence (100%) and lift (2.8) referred to death during the first two months of treatment (intensive phase) of men, aged 35 to 59 years, new TB cases or relapse, diagnosed in hospital (rules 1 to 4). Rule 5 has the greater support (1.4%) and refers to men, aged 35 to 59 years, Black/Mixed-race skin color with HIV-negative test who died in the intensive treatment phase. Among the other rules that included death during the first two months, two also had men, new case, and were treated at a PHC unit (rules 6 and 7). Rule 6 also included low education and HIV-negative test, whereas rule 7 also included young age. Rules 8 and 9 referred to women, who were diagnosed at a hospital. The latter rules differed because rule 8 included relapse, and rule 9 included young age. Rules that indicated the diagnosis of TB near the time of death (rules 10 to 15) also included patients diagnosed in hospital and with a laboratory test not performed (smear test or HIV). Male, aged 35 years or more and new case occurred more frequently in such rules. The two rules that included deaths during the maintenance treatment phase or at most within one year after diagnosis (rules 16 and 17) also had men that returned after default and an HIV-negative test. Finally, rule 18 referred to a more vulnerable to death profile consisting of Black or Mixed-race men and lower educational level, who returned after default.

**Table 2:** Support, confidence and lift of top association rules selected by experts for deaths caused by tuberculosis (TB). Rio de Janeiro, Brazil, 2009 to 2012.

Antecedent	Support (%)	Confidence (%)	Lift	n
Consequent - deaths from TB				
(1) Age = 35-59 years, survival time = 0-60 days, education = ignored, skin color = White, sputum smear test = tested	0.8	100.0	2.8	15
(2) Sex = male, age = 35-59 years, survival time = 0-60 days, education = ignored, skin color = ignored, sputum smear test = not tested	0.7	100.0	2.8	14
(3) Sex = male, age = 35-59 years, survival time = 0-60 days, skin color = Black/Mixed-race, HIV test = negative	1.4	71.4	2.0	26
(4) Sex = male, age = 35-59 years, survival time = 0-60 days, education = ignored, HIV test = not tested, new case, sputum smear test = tested, hospital	0.7	100.0	2.8	14
(5) Sex = male, survival time = 0-60 days, relapse, sputum smear test = tested	0.6	100.0	2.8	11
(6) Sex = male, survival time = 0-60 days, education = 0-4, HIV test = negative, new case, Municipal Health Care Center	0.6	90.9	2.5	11
(7) Sex = male, age = 16-34 years, survival time = 0-60 days, new case, Municipal Health Care Center	0.7	86.7	2.4	14
(8) Sex = female, survival time = 0-60 days, relapse, hospital	0.5	90.9	2.5	10
(9) Sex = female, age = 16-34 years, survival time = 0-60 days, hospital	1.0	78.3	2.2	18
(10) Age = 35-59 years, survival time = notified after death, sputum smear test = not tested, hospital	0.6	83.3	2.3	11
(11) Age = 35-59 years, survival time = notified after death, HIVv test = not tested, hospital	0.6	83.3	2.3	11
(12) Survival time = notified after death, skin color = Black/Mixed-race, hiv test = not tested, sputum smear test = not tested, hospital	0.6	78.5	2.2	11
(13) Sex = male, survival time = notified after death, new case, sputum smear test = not tested, hospital	0.8	77.8	2.2	15
(14) Sex = male, survival time = notified after death, HIV test = not tested, new case, sputum smear test = not tested, hospital	0.7	76.5	2.2	14
(15) Age = 60 years or older, survival time = notified after death, new case, sputum smear test = not tested, hospital	0.6	71.4	2.0	11
(16) Sex = male, survival time = 61-180 days, HIV test = negative, return after default, hospital	0.6	83.3	2.3	11
(17) Sex = male, survival time = 181-365 days, HIV test = negative, return after default, sputum smear test = tested	0.6	83.3	2.3	11
(18) Sex = male, education = 0-4, skin color = Black/Mixed-race, return after default	1.3	72.7	2.0	24

Rules related to deaths from TB-HIV/AIDS coinfection (Table 3) had maximum support of 0.8%, maximum lift of 6%, and maximum confidence of 12.5%. The rules with a higher lift (≥ 5.4) referred to women not tested for HIV (rules 19 to 21), new TB cases diagnosed in hospital (rule 19), aged 35 to 59 years (rule 20), with educational level ignored (rule 21). Three other rules (rules 22, 23, 24) included being treated in PHC and not presenting a sputum smear test. Rules 25 and 26 referred to deaths that occurred during treatment. The last rule (27) included only age (16 to 34 years). For deaths from AIDS without TB, we selected six rules (Table 3, rules 28 to 33) which presented maximum support of 0.6%, maximum lift of 1.9%, and maximum confidence of 2.7%. The combinations of items were more varied. Only one rule selected included male sex, which differs from deaths from TB-HIV/AIDS coinfection, in which we only selected rules that included women.

**Table 3:** Support, confidence and lift of top association rules selected by experts for AIDS deaths with and without tuberculosis (TB). Rio de Janeiro, Brazil, 2009 to 2012.

Antecedents	Support (%)	Confidence (%)	Lift	n
Consequent - death from AIDS with TB				
(19) Sex = female, HIV test = not tested, new case, hospital	0.5	12.5	6.0	10
(20) Sex = female, age = 35-59 years, HIV test = not tested	0.5	12.5	6.0	10
(21) Sex = female, education = ignored, HIV test = not tested	0.6	11.2	5.4	11
(22) Age = 35-59 years, sputum smear test = not tested, Municipal Health Care Center	0.5	7.0	3.4	10
(23) Survival time = 61-180 days, HIV test = not tested, new case	0.5	6.7	3.2	10
(24) Skin color = Black/Mixed-race, sputum smear test = not tested, Municipal Health Care Center	0.5	5.8	2.8	10
(25) HIV test = not tested, sputum smear test = not tested, Municipal Health Care Center	0.6	4.7	2.3	11
(26) Survival time = 61-180 days	0.8	4.4	2.1	15
(27) Age = 16-34 years	0.8	4.1	1.9	15
Consequent - death from aids without TB				
(28) Age = 16-34 years	0.5	2.7	1.9	10
(29) Age = 35-59 years, HIV test = not tested	0.6	2.7	1.9	11
(30) Sex = male, HIV test = not tested, Sputum smear test = not tested	0.5	2.7	1.9	10
(31) Age = 35-59 years, Municipal Health Care Center	0.5	2.7	1.9	10
(32) Skin color = Black/Mixed-race, Municipal Health Care Center	0.6	2.5	1.8	11
(33) HIV test = not tested, new case, sputum smear test = not tested	0.5	2.3	1.6	10

Table 4 shows the four rules selected for deaths from external causes. Three of them included young men (rules 34 to 36) who died after treatment. The rules selected had maximum support of 0.7%, a maximum lift of 9.9%, and maximum confidence of 84.6%. Ten of the twelve chosen rules for deaths from other causes presented 100% confidence and a 1.9 lift (rules 38 to 49). We selected 12 rules related to deaths from other causes (Table 4). Such rules had maximum support of 2% and a maximum lift of 1.9. Ten of them had 100% confidence. Most rules included deaths one year after the diagnosis, older people, and those diagnosed in PHC. Differently from the observations in the other groups, the category white predominated when the rule selected included skin color.

**Table 4:** Support, confidence and lift of top association rules selected by experts for deaths from external causes and other causes. Rio de Janeiro, Brazil, 2009 to 2012.

Antecedents	Support (%)	Confidence (%)	Lift	n
Consequent - death from external causes				
(34) Sex = male, age = 16-34 years, survival time = over 365 days, education = 9-12, new case	0.6	84.6	9.9	11
(35) Sex = male, age = 16-34 years, survival time = over 365 days, education = 5-8, skin color = Black/Mixed-race, HIV test = not tested	0.7	80.0	9.4	12
(36) Sex = male, age = 16-34 years, survival time = 181-365 days, sputum smear test = tested, Municipal Health Care Center	0.5	52.6	6.2	10
(37) Sex = male, skin color = Black/Mixed-race, return after default, Municipal Health Care Center	0.6	20.4	2.4	11
Consequent - death from other causes				
(38) Age = 60 years or older, survival time = over 365 days, skin color = White, HIV test = not tested, sputum smear test = not tested	2.0	100.0	1.9	37
(39) Age = 60 years or older, survival time = over 365 days, HIV test = not tested, new case, sputum smear test = not tested, Municipal Health Care Center	1.8	100.0	1.9	33
(40) Survival time = over 365 days, education = over 12, skin color = White, HIV test = not tested	0.7	100.0	1.9	13
(41) Sex = female, age = 60 years or older, survival time = over 365 days, HIV test = not tested, Municipal Health Care Center	1.1	100.0	1.9	20
(42) Sex = male, age = 60 years or older, survival time = over 365 days, skin color = White, HIV test = not tested, new case, sputum smear test = not tested, Municipal Health Care Center	0.8	100.0	1.9	15
(43) Sex = male, age = 60 years or older, survival time = over 365 days, skin color = White, HIV test = negative, new case, sputum smear test = not tested, Municipal Health Care Center	0.6	100.0	1.9	11
(44) Sex = male, age = 35-59 years, survival time = over 365 days, skin color = White, HIV test = negative, new case, sputum smear test = tested, Municipal Health Care Center	0.8	77.8	1.5	15
(45) Sex = male, age = 35-59 years, survival time = over 365 days, skin color = Black/Mixed-race, new case, sputum smear test = tested, Municipal Health Care Center	1.6	75.0	1.4	30
(46) Age = 60 years or older, survival time = 181-365 days, skin color = Black/Mixed-race, HIV test = not tested	0.7	100.0	1.9	13
(47) Age = 60 years or older, education = 5-8, HIV test = negative, TB clinic	0.5	100.0	1.9	10
(48) Age = 35-59 years, HIV test = negative, TB clinic, sputum smear test = not tested	0.6	100.0	1.9	11
(49) Survival time = over 365 days, education = 0-4, HIV test = not tested, relapse	0.5	100.0	1.9	10

## Discussion

Using the Apriori algorithm enabled us to identify various mortality patterns related to different patient profiles. The TB profiles differed according to the cause of death and considered the survival time after TB diagnosis. Overall, TB deaths were associated with adult men with shorter survival times. Deaths from AIDS with TB occurred within the treatment period and were more associated with women. In contrast, deaths from AIDS without mention of TB were associated with young men. Deaths from external causes occurred later, one year after diagnosis, and young men were the most common characteristic found in this profile. Deaths from other causes, related to longer survivals, were associated with older adults and white skin color.

Even more important than the identification of those relevant characteristics associated with the underlying cause of death was the identification of patient profiles. Studies using traditional regression models show that all TB mortality causes were associated with old age ^29^ ^,^ ^30^, male sex ^31^, low socioeconomic status ^32^ ^,^ ^33^, presence of comorbidities ^30^ ^,^ ^34^ ^,^ ^35^, abuse of alcohol or other illicit drugs ^36^ ^,^ ^37^ and previous treatment for TB ^30^ ^,^ ^36^ ^,^ ^38^. However, one limitation of such traditional approaches is that they make it hard to identify specific subgroups of more vulnerable people for unfavorable outcomes. Traditional studies on risk factors for treatment outcomes generally produce measures of association for each factor individually, after adjusting for others. Association rules, on the other hand, identify which factors - whether individually or in combination - are most relevant in defining profiles with high risks of distinct outcomes, including both frequent and rare patterns. In our study, using association rules, we identified profiles of patients that could benefit from greater consideration in health care services, aiming at better outcomes in their TB treatment.

Death from TB is a preventable event ^39^. However, more than one-third of people who died with pulmonary TB had this condition as the underlying cause of death, a result that was also observed in a previous study ^11^. Among TB deaths, the shorter survival time was highlighted as well as those patients diagnosed at death. Men, especially adults and older adults, were the most affected. Enhancing the management of respiratory symptomatic individuals in PHC would promote a more timely diagnosis of TB and, consequently, reduced mortality.

Shorter survival time may indicate a barrier to access to timely treatment. This hypothesis is reinforced by the lack of HIV and sputum smear tests, leading to the death of new TB cases occurring even before the diagnosis of the disease ^8^ ^,^ ^40^.

Diagnostic delay may increase disease severity and lead to a higher risk of death ^41^. Diagnostic delay is a function of the interval time that comprises the onset of symptoms, decision to seek care, obtaining medical care, and receiving diagnosis of the disease. Failure in diagnosis accounts for an important portion of the delay to treatment ^42^. Factors associated with the subject’s vulnerability that influence the outcome add to explanations for early death. Malnutrition ^43^ and poor performance status ^44^ were associated with early death in hospitalized people. The presence of some comorbidities in HIV-negative subjects with pulmonary TB in a Brazilian study highlighted those related to infectious diseases, those related to blood diseases, immunological disorders, smoking, and lung diseases ^45^.

The presence of young men and men with low levels of education treated at a Municipal Health Center was notable in deaths occurring in the 2-month intensive phase of TB treatment. The decentralization of TB treatment and diagnosis to PHC is one of the measures against the disease in Brazil ^3^. Moreover, the male sex is related to lower use of health care services, considering that men and young people are not regular visitors to health care facilities. We found a relevant proportion of young men dying from TB with markers of low access to health care. On the other hand, some young women with recurrent TB fell in the group that died from TB less than two months after diagnosis. They composed a group of subjects diagnosed in general hospitals, which may have contributed to the occurrence of an unfavorable outcome.

Differently from new TB cases, deaths from TB in HIV-negative men who defaulted treatment occurred during the maintenance treatment phase (60-180 days) or after the end of treatment, but before one year after diagnosis. It is known that treatment default is related to an increased risk of dying from TB and is often related to socially vulnerable people ^46^. However, the association rules elicited here did not include deprived subjects, such as Black/Mixed-race skin color subjects with low education levels. This fact may indicate that not only these groups had the possibility of a worse prognosis.

For deaths from TB-HIV coinfection, there was a predominance of females, without sputum smear and HIV tests performed, Black or Mixed-race skin color, aged 16 to 59 years, treated at a Municipal Health Care Center and dying within the treatment period. Women of reproductive age visit health care facilities more frequently than men, which should have helped the earlier detection of TB. However, women are not a predominant subject in TB and AIDS ^47^, which may have contributed to misdiagnosis at PHC facilities, differently from expectation. On the other hand, men were predominant in deaths from AIDS without mention of TB. They were young adults, Black/Mixed-race skin color, treated at a Municipal Health Care Center, who did not undergo diagnostic tests for HIV and TB, and there was no association with the survival time after TB treatment initiation. They probably had undiagnosed HIV infection, and both TB and HIV disease should be detected and treated in due time. In Brazil, every person diagnosed with TB must be tested for HIV ^3^. The loss of opportunity to detect HIV may have contributed to the occurrence of preventable deaths.

In deaths from external causes, we note young men with high school education or Blacks with low education, whose deaths occurred later after the end of TB treatment. TB is associated with poverty, which, in turn, is also associated with violence. Young men compose a vulnerable group in this regard in Rio de Janeiro ^48^ ^,^ ^49^. Also, Mixed-race men were more likely to die from external causes (28%) than Black, Yellow, Indigenous, and White men (8%). Deaths from external causes are associated with young men living in areas of social vulnerability in large urban cities ^50^.

It is widely known that poor living conditions increase TB morbidity and hinder access to health care services. Black and Mixed-race people had a lower probability of cure, suggesting untimely diagnosis and inadequate treatment ^51^. Therefore, it is essential to implement social protection policies that address systemic racism, stigma, and access barriers in order to reduce the current structural inequality and promote equity.

Studies on multiple causes of death involving individuals with TB report that the main underlying causes - when not TB itself - are AIDS, neoplasms, circulatory diseases and respiratory system diseases ^11^ ^,^ ^52^. In our study, deaths from other causes occurred one year after TB diagnosis and were associated with older adults without an HIV test and to white people with high education, the latter a group typically considered less vulnerable to TB. These findings are consistent with the understanding that preexisting diseases and a declined immune response can explain the onset of TB in older individuals. This also might highlight a key concern, as TB may worsen existing conditions and increase the risk of death, even after successful treatment. Patients treated at TB clinics, a health care service with a secondary level of complexity for TB care, may indicate difficulty in treatment management, increasing the risk of death even after the cure of the TB episode ^9^. On the other hand, Black/Mixed-race older adults died earlier, between six months and one year after diagnosis, and TB could be considered as a contributing cause of death.

The use of metrics such as lift and confidence was essential to choose rules that indicated specific profiles, instead of support, more indicated to highlight the most common and general patterns ^12^. The lift measures the dependency between items in a data set and the highlighted rules had values ​​above two in most. Although some rules have high values of confidence and lift, they can be considered rare and, therefore, some consider them to be of little use ^53^. However, since TB is a preventable and curable disease, understanding the factors associated with death, combined or isolated, can improve health care initiatives.

The findings of this study may also benefit other populations and geographical contexts, considering there are no significant differences in the profile of TB-related deaths nationwide. This is further supported by the fact that recommendations for TB prevention, diagnosis, and treatment are standardized throughout the country ^3^. Additionally, the study’s methodology can be used to assist with the diagnosis and outcome analysis of specific patient groups, such as individuals with extrapulmonary TB, drug-resistant TB, HIV, or diabetes. Furthermore, health authorities can use this tool to guide targeted strategic measures aimed at increasing the effectiveness of health care.

The results of this study highlight that prevention strategies within PHC, in close collaboration with surveillance, can be expanded or strengthened at the local level. These strategies can be applied to different patient groups to varying degrees and include contact investigation, expansion of TB preventive treatment, active case search for respiratory symptomatic individuals, strengthening of intersectoral programs to reduce treatment interruption, ongoing education for health care professionals, development of health education initiatives for the community using social media and accessible communication.

This study is subject to several key limitations. The time period analyzed may not fully account for recent changes, such as data enhancements in the SINAN-TB database and improvements in health care in Rio de Janeiro, both of which could influence the mortality profile. An example is the lack of specific variables for incarcerated and homeless populations, which prevents the identification of other patient profiles. The findings do not account for the potential impact of the RMT-TB, which has influenced early diagnosis and detection of rifampicin resistance since its implementation in 2014. The extended effects of the COVID-19 pandemic beyond 2021 were not considered, a factor that has impacted health care services and altered global mortality trends for individuals with TB. Furthermore, a limitation of the study is that some individuals had a passive follow-up period of only one year, which may have constrained the observation of later-onset mortality.

Even though the data are not recent, it is worth noting that despite the availability of RMT-TB and improvements in TB care and surveillance in the city of Rio de Janeiro over the years, the cure rate has remained stable. Meanwhile, the mortality rate showed a slight increase between 2012 and 2014, with a gradual reduction until 2016, followed by a plateau, except during the pandemic period ^2^. Furthermore, between 2012 and 2023, the case profile did not show any significant changes, and the rate of laboratory confirmation decreased. This suggests that, even when referring to the 2009-2012 period, the improvements mentioned likely did not lead to substantial changes in the mortality rate.

Another limitation of this study is the non-inclusion of other variables that may influence mortality, such as quality of care, multidrug-resistant TB, and comorbidities. Furthermore, the study’s reliance on routine data from health information systems is a limitation, as these data are subject to the quality of completion by professionals. As a result, skin color and education data had high percentages of missing values. To minimize possible bias in excluding records with missing values, a category of non-information was added to these variables. Possible errors in the linkage process could also be noted, such as pairs of records that were not found. However, the conservative approach of considering doubtful links as non-pairs minimizes potential bias in the association measures (e.g., odds ratio and relative risk), and, by extension, in the findings of this study’s association rules ^23^.

Another limitation was the low support of the rules, highlighting patterns with few records. Association rule algorithms can find associations that other methods, such as regression models, do not easily detect. However, there is still no technique for statistical validation of the rules found, whose selection is based empirically using metrics and expert judgment. However, this aspect can only be evaluated in a future study that consists of databases even larger than the one used in the present study, and that repeated the health care system conditions observed in the analyzed period.

## Conclusion

The Apriori algorithm was able to extract relevant knowledge from secondary databases, identifying mortality patterns, which indicated distinctive subgroups of subjects, who can benefit from specific measures, with a positive impact on TB control. The Apriori algorithm can be used for knowledge acquisition in the public health care sector, to monitor actions, and to support the surveillance and decision-making process.

## Data Availability

The research data are not available.
